# IL-18 and IL-18BP: A Unique Dyad in Health and Disease

**DOI:** 10.3390/ijms252413505

**Published:** 2024-12-17

**Authors:** Daniela Novick

**Affiliations:** Molecular Genetics, The Weizmann Institute of Science, Rehovot 7610001, Israel; daniela.novick@weizmann.ac.il

**Keywords:** IL-18, IFN-γ, IL-18BP, Tadekinig alfa, antidote, antagonist, agonist, checkpoint, CAR T, MAS, HLH, COVID-19, NLRC4, XIAP, inflammasome, double-edged sword

## Abstract

Interleukin-18 (IL-18) serves a dual function in the immune system, acting as a “double-edged sword” cytokine. Depending on the microenvironment and timing, IL-18 can either drive harmful inflammation or restore immune homeostasis. Pathologies characterized by elevated IL-18, recently proposed to be termed IL-18opathies, highlight the therapeutic potential for IL-18 blockade. IL-18 Binding Protein (IL-18BP) is one of only four natural cytokine antagonists encoded by a separate gene, distinguishing it from canonical soluble receptors. IL-18BP’s exceptionally high affinity and slow dissociation rate make it an effective regulator of IL-18, essential for maintaining immune balance and influencing disease outcomes, and positions IL-18BP as a promising alternative to more aggressive treatments that carry risks of severe infections and other complications. Tadekinig alfa, the drug form of IL-18BP, represents a targeted therapy that modulates the IL-18/IL-18BP axis, offering a safe adverse-effect-free option. With orphan drug designation, Phase III clinical trial completion, and seven years of compassionate use, Tadekinig alfa holds promise in treating autoimmune and inflammatory diseases, cancer, and genetically linked disorders. Levels of IL-18, free IL-18 and IL-18BP, may serve as biomarkers for disease severity and therapeutic response. Given its pivotal role in immune balance, the IL-18/IL-18BP dyad has attracted interest from over ten pharmaceutical companies and startups, which are currently developing innovative strategies to either inhibit or enhance IL-18 activity depending on the therapeutic need. The review focuses on the features of the dyad members and screens the therapeutic approaches.

## 1. Introduction

Inflammation, an ancient response of innate immunity, is triggered by infection and by tissue damage with the goal of eliminating these threats. This response is followed by acute inflammatory processes that set the stage for adaptive immunity within a precise time frame and microenvironment. The induction of various intercellular communication proteins, known as interleukins and cytokines, including IL-18 (Landy et al., 2024) [1] is crucial for this process. However, uncontrolled inflammation can lead to excessive tissue damage, chronic inflammation, autoimmunity, fibrosis, and eventual organ failure. To prevent such detrimental outcomes, a mechanism that includes the feedback regulation of the various cytokine pathways is required to resolve inflammation and to restore homeostasis. This regulatory role is played by naturally occurring soluble receptors and binding proteins. Natural soluble receptors (Novick & Rubinstein, 2007) [2] that are the counterparts of cell surface receptors’ extracellular domain, such as those of IL-1, TNF, and IFNs, are the canonical receptor antagonists. Natural binding proteins, with a similar function, are encoded by separate genes and are rare, unique, and attractive candidates for therapeutic development. One such example is IL-18BP, the highly effective antagonist of IL-18 (Novick et al., 1999) (Fauteux-Daniel et al., 2023) [3,4]. This review focuses on the dual role of IL-18 and the crucial function of its unique partner, IL-18BP, in regulating this cytokine to keep it in the normal range, and the strategies pursued by over ten pharmaceutical companies and startups to target IL-18 and IL-18BP in autoimmune diseases, rare mutations, cytokine storm, cancer, and COVID-19.

### 1.1. Key Features of IL-18

1. **Origin and Family**
  - Originally named IFN-γ-inducing factor, IL-18 is a member of the IL-1 family.
  - It bridges innate and adaptive immunity, regulated by the cytokine it induces.
2. **Dual Role: Beneficial or Harmful**
  - IL-18 acts as a “double-edged sword” cytokine.
  - Depending on the microenvironment, it can either promote inflammation or restore immune homeostasis.
3. **Th1 or Th2 Functionality**
  - Mainly a Th1-associated pro-inflammatory cytokine but also a Th2 cytokine, depending on the microenvironment.
4. **Standby Mechanism**
  - IL-18 does not require de novo synthesis.
  - In its inactive pro-form is ready to be activated by caspase-1.
5. **Cytokine Signature**
  - IL-18 induces IFN-γ as its signature response, activates NK cells and cytotoxic T cells and regulates over 1000 genes.
6. **IL-18 Receptor and Signaling Pathway**
  - IL-18 signals through an evolutionarily conserved receptor composed of a ligand-binding chain and a signal-transducing chain.
  - IL-18 receptor is evolutionarily conserved and belongs to the Toll-like pattern recognition and induction of innate immune responses family of receptors (TLRs).
  - IL-18 signaling involves MyD88, IRAKs, and TRAF-6 to trigger NFκB activation and proinflammatory cascades.
7. **Role in Diseases**
  - Involved in autoimmune diseases, inflammation, inborn disorders, infections (e.g., severe COVID-19), and cancer.
8. **Biomarker in “IL-18opathies”**
  - A biomarker in IL-18opathies characterized by exceptionally elevated IL-18 levels that facilitate differential diagnosis in related disorders.
9. **Cancer and CAR-T Cells**
  - IL-18 acts as a checkpoint biomarker in cancer.
  - IL-18-engineered CAR-T cells enhance tumor-killing in solid tumors, especially in PD-1 blockade therapies.
10. **Cytokine Storm Player**
  - IL-18 is a major contributor to cytokine storms linked to viral infections, autoimmune diseases, cancer, and CAR-T therapies.
  - IL-18BP is being explored as a rescue treatment.
11. **Safer Blockade Option**
  - Blocking IL-18 seems a safer option than inhibiting its family member, the master cytokine, IL-1, as prolonged IL-1 inhibition risks severe infections.

### 1.2. Key Features of IL-18BP

1. **Unique Antagonist**
  - IL-18 Binding Protein (IL-18BP), unlike canonical soluble receptors, is a rare example of a cytokine antagonist encoded by a separate gene.
2. **Binding Specificity and Variants**
  - IL-18BP is a glycosylated protein that binds mature IL-18 and not the inactive pro-IL-18.
  - In humans, IL-18BPa is the most abundant and active of its four splice variants.
3. **Exceptional Affinity**
  - IL-18BPa has an exceptionally high affinity for IL-18 and a slow dissociation rate (K_off_), features that ensure stable dyad complexes, making it an effective regulator of IL-18 signaling and a promising therapeutic agent.
4. **Pharmacokinetics**
  - Unlike antibodies with a half-life of 3–4 weeks, IL-18BP’s shorter half-life (34–40 h) allows rapid cessation, enabling timely IL-18 activity when urgently needed for immune defense.
5. **Homeostatic Regulation**
  - The balance between IL-18 and IL-18BPa, particularly free IL-18 levels, is regulated by IFN-γ in a feedback loop.
  - This balance is critical for maintaining homeostasis and determining disease outcomes.
6. **Life-Threatening Deficiency**
  - IL-18BP deficiency can be fatal if untreated, highlighting its critical role in health and disease.
7. **Therapeutic Applications**
  - Tadekinig alfa, a recombinant IL-18BPa, is a life-saving drug for children with inborn IL-18 overexpression and organ failure.
  - A Phase III clinical study has been completed and the treatment has remained in compassionate use for seven years with no reported adverse effects.
  - Proven beneficial in Still’s disease, it also shows promise for cytokine storms caused by viral infections, cancer, and CAR-T therapy.

## 2. IL-18

### 2.1. IL-18 Is an IFN-γ Inducing Factor (IGIF)

IL-18, initially named interferon-g inducing factor (IGIF) (Nakamura et al., 1989) [5], is an IL-1 family member and a cytokine that links innate and adaptive immunity. The IL-1 family of cytokines and receptors is unique in immunology and shares functional similarities with the Toll-like receptor (TLR). Innate immune mechanisms are essential for survival by the vast majority of living organisms while less than 5% utilize T cell and B cell functions. Innate immunity is manifested by inflammation aimed at host defense, but when uncontrolled, it can be harmful to survival. This may explain the evolutionary emergence of IL-18BP, IL-18’s potent natural antagonist and the other partner in the dyad.

### 2.2. IL-18 Is of a Dual Nature

IL-18’s dual nature, functioning as either beneficial or detrimental, and its classification as a Th1 or Th2 cytokine, depend on the microenvironment in which it operates. Other members of this “double-edged sword” group of cytokines (Marx, 1988) [6] include TNF (Aggarwal, 2003) [7], IFN-γ (Zaidi & Merlino, 2011) [8], IL-6 (Blanchard et al., 2009) [9], IL-25 (Monteleone et al., 2010) [10], Type I IFNs (Snell et al., 2017) [11], and cytokines participating in sepsis (Chaudhry et al., 2013) [12] and in tuberculosis (Etna et al., 2014) [13]. In a healthy immune system, IL-18 plays a beneficial role in the host defense against infections and in the regulation of immune responses aimed to maintain homeostasis. However, in a disease setting, such as autoimmune diseases, inborn disorders, or chronic inflammatory conditions, IL-18 can become dysregulated, leading to excessive inflammation which may result in a cytokine storm, tissue damage, organ failure, and even death. Thus, tight regulation of IL-18 is essential.

### 2.3. IL-18 Is a Th1 and a Th2 Cytokine

Traditionally, IL-18 has been associated with Th1 immune responses because it induces the production of IFN-γ, a hallmark cytokine of Th1 cells. In Th1-dominated immune responses, IL-18 enhances the activity of NK cells and cytotoxic T cells, e.g., to combat intracellular pathogens, fight cancer cells, and trigger autoimmune diseases (Nakanishi et al., 2001) (Steinman, 2007) (Novick et al., 2013) [14,15,16]. IL-18 acts on non-polarized T cells, NK cells, NKT cells, B cells, dendritic cells, and macrophages. In the presence of IL-12, shown to increase the expression of the transducing chain of the IL-18 receptor, IL-18 induces the production IFN-γ. It induces chemokines and cell adhesion molecules, stimulates inflammatory cytokine secretion such as IL-1 and TNF-a, and enhances NK-cell cytotoxicity. Yet in a different microenvironment IL-18 can promote Th2 immune response which is characterized by the production of cytokines such as IL-4, IL-5, and IL-13, and is involved in allergic reactions, asthma, and parasitic infections. IL-18 without IL-12 but with IL-2 induces Th2 cytokine production. IL-18 with IL-3 induces mast cells and basophils to produce IL-4 and IL-13 (Yasuda et al., 2019) [17]. IL-18 may also promote the differentiation and activation of regulatory T cells (Tregs), and thus suppress immune responses aiming to maintain tolerance (Alvarez et al., 2023) [18]. Cancer is yet an additional example in which IL-18’s dual nature is exhibited. In certain types of cancer, IL-18 has been shown to be anti-tumorigenic and to enhance immunotherapy and chemotherapy. In other types of cancer IL-18 was tumor-promoting (Fabbi et al., 2015) (Ihim et al., 2022) [19,20]. It is of no surprise that with such a vast scope of activities and depending on its immediate vicinity, IL-18 is tightly regulated by its partner in the dyad, IL-18BP.

### 2.4. Pro-IL-18: Always on Standby

IL-18 does not require a de novo synthesis. The inactive pro-IL-18 is always on standby ready to be processed to its active form upon the right trigger. Thus, in response to a pathogen or stress-associated stimuli, pro-IL-18 is proteolytically cleaved to its bioactive form by the cytosolic inflammasome component, caspase-1. Caspase-1 also activates the pore-forming Gasdermin D (GSDMD), enabling the secretion of the leaderless cytokine, IL-18 (Xia et al., 2021) [21]. This process initiates pyroptosis, a lytic programmed cell death, aiming at rapid clearance of various bacterial, viral, fungal, and protozoan infections.

### 2.5. IL-18 Dictionary of Interactions

IL-18 had been included in a novel dictionary of immune responses to cytokines at a single-cell resolution. This dictionary is an in vivo collection of 386,703 single-cell transcriptomic profiles of more than 17 immune cell types in response to each of the 86 cytokines (>1400 cytokine–cell type combinations) demonstrated in mouse lymph nodes. Most cytokines induce highly cell-type-specific responses. Based on this dictionary and using gene expression data, a software named Immune Response Enrichment Analysis (IREA) (GSE202186), was developed, and cytokine activities and immune cell polarization were assessed. IFN-γ was confirmed to be IL-18’s signature and NK cells its main target. More than 20 cell types were identified and differentially expressed genes (DEGs) in response to cytokine treatment in each cell type were computed, aiming to compose cytokine signatures. In addition, a map that quantified transcriptomic changes upon cytokine treatment was created. Of note is that IL-18 triggered the upregulation of more than 1000 genes, an order of magnitude more than demonstrated for other cytokines. The IL-18-induced state was strongly enriched in a variety of processes such as the induction of *Myc* (which controls growth and proliferation), maturation of myeloid cells, recruitment of dendritic, cytotoxicity, and regulators of differentiation (*Kit* and *Batf*). The value of this dictionary is indispensable in therapeutically applied cytokines, including IL-18. Indeed, this dictionary was applied to reveal cytokine networks in tumors following immune checkpoint blockade therapy such as PD-1 blockade (Cui et al., 2024) [22]. This high-resolution dictionary provides empirical evidence for IL-18’s role in immune response and substantiates its uniqueness and importance.

### 2.6. IL-18: A Non-Master Cytokine

IL-18 is not a master cytokine by itself but is a member of a master cytokine family, IL-1, and is regulated by the master cytokine IFN-γ. While IL-1 exerts broad and systemic effects on immune and inflammatory pathways, IL-18’s actions are more specific and context-dependent. Therefore, blocking IL-18 signaling is less likely to disrupt the entire immune system, making it a more targeted therapeutic approach with potentially fewer adverse effects. This distinction emphasizes the therapeutic promise of targeting IL-18 rather than IL-1 in the relevant immune-mediated disorders.

### 2.7. IL-18 Regulation

IL-18 is regulated by the cytokine it induces, the master cytokine IFN-γ (Hurgin et al., 2002) [23]. So far, IFN-γ is IL-18’s only known regulator. IFN-γ modulates IL-18 production and activity via a negative feedback mechanism, and thereby fine-tunes immune responses and inflammatory processes. In an excess of IFN-γ, IL-18’s natural antidote, IL-18BP, is induced with the aim to restore homeostasis.

### 2.8. Cells Producing IL-18

IL-18 is produced by both hematopoietic and non-hematopoietic cells, such as T cells, NK cells, dendritic cells, macrophages, endothelial cells, keratinocytes, intestinal epithelial cells, microglial cells, and synovial fibroblasts (Yasuda et al., 2019) [17].

### 2.9. IL-18 Deficiency (Knockout Models)

IL-18 knockout (KO) mouse models provide insights into the multifaceted roles of IL-18 in immune regulation, inflammation, autoimmune diseases, infectious diseases, metabolic disorders, and cancer. IL-18 KO mice showed reduced levels of IFN-γ and lower NK cell activity, leading to a diminished ability to combat pathogens and protect against cancer. IL-18 KO mice showed reduced severity in models of autoimmune diseases, e.g., arthritis, lupus, and inflammatory bowel disease (IBD) pointing to the deleterious effect of an excess of IL-18 in human diseases. IL-18 KO mice on high-fat diets have been shown to develop more severe obesity and insulin resistance, indicating the role of IL-18 in metabolic syndrome. IL-18 KO mice showed cognitive impairment and depressive-like behavioral changes, demonstrating the involvement of IL-18 in psychiatric and neurologic conditions. Cancer, metabolism, and brain disorder-related genes referring to IL-18 are listed in the review by Yamanishi K et al., 2023 (Yamanishi et al., 2023) [24]. These models (Netea et al., 2006) [25] reveal potential therapeutic targets in the corresponding human pathologies and contribute to the development of novel treatments. 

## 3. IL-18 Receptor

### 3.1. IL-18 Receptor Composition

IL-18 receptor (IL-18R) is composed of two chains: the ligand binding chain, IL-18R alpha (IL-1R5), and the IL-18 receptor beta accessory chain, IL-18Rap (IL-1R7). IL-18 first binds to IL-18Ra, followed by the binding of IL-18Rap, and this high affinity trimeric complex initiates signal transduction. The Toll-IL-1-Receptor (TIR) intracellular domains of the receptor chains come into proximity and a cascade of sequential events is initiated. It involves the recruitment of MyD88, the four IRAKs, and the TNF receptor activating factor-6, followed by the degradation of IκB, release of NFκB, and activation of a proinflammatory process, e.g., the release of pro-inflammatory cytokines (Rex et al., 2020) (Tsutsumi et al., 2014) [26,27]. The IL-18 receptor is most prominently expressed by NK cells and activated memory T cells. Hoshino K et al. (Hoshino et al., 1999) [28] have generated IL-18Rα-deficient mice and observed a lack of Th1 response upon IL-18 stimulation in these ligand-binding chain knockout mice.

### 3.2. IL-18 Receptor: Evolutionary Conserved

The IL-18 receptor is conserved throughout evolution. It belongs to the IL-1 family of receptors and shares functions with the TLR family. These receptors are pattern recognition receptors responsible for pathogen recognition and the induction of innate immune responses and as such, link IL-18 with innate immunity. Each member of these families contains a 50-amino-acid cytoplasmic TIR domain, highly homologous to the ancient Toll gene found in Drosophila and other insects (Tsutsumi et al., 2014) [27].

### 3.3. IL-18 Receptor Expression

Most cells express the ligand binding chain of the receptor IL-18Ra, but not all cells express the transducing chain IL-18Rb. IL-18R is mainly expressed in hematopoietic cells such as CD4^+^ NKT cells, mast cells, basophils, and T cells, with the highest expression observed in NK cells, driving its differentiation and activation (Nakamura et al., 2000) [29]. The expression of IL-18R on Th1 cells and B cells is mainly driven by IL-12 (Yoshimoto et al., 1998) (Sareneva et al., 2000) (Rex et al., 2020) (Tsutsumi et al., 2014) [26,27,30,31].

### 3.4. IL-18 Receptor Binding Sites

IL-18 ligand binding sites to its receptor and to the IL-18 binding protein are formed upon conformational changes created following pro-IL-18 cleavage by caspase-1. A study by Dong et al. has shown why this cleavage is necessary for mature IL-18’s inflammatory activity (Dong et al., 2024) [32].

### 3.5. IL-18 Affinity to Its Receptor

The affinity of IL-18 to its ligand-binding cell surface receptor chain is 18.5 nM. The affinity rises to 0.4 nM when the accessory chain joins to form a signal transducing functional triple complex (Wu et al., 2003) (Tsutsumi et al., 2014) [27,33]. This affinity is comparable to the affinity of IL-18 to its natural antagonist, IL-18BP (Kim et al., 2000) (Girard et al., 2016) [34,35].

## 4. IL-18 in Health and Disease

### 4.1. IL-18 and Free IL-18 Levels in Healthy Individuals and in a Pathology

The level of IL-18 in healthy people is around 100 pg/mL. In IL-18opathies, the level may rise 10–10,000 fold, e.g., in sepsis, Still’s disease, hemophagocytic lymphohistiocytosis (HLH), or macrophage activation syndrome (MAS) (Novick et al., 2001) (Mazodier et al., 2005) (Girard et al., 2016) (Novick et al., 2013) [15,34,36,37]. Most of the circulating IL-18 is in a tight complex with its antagonist, IL-18BP; therefore, in healthy people, the blood concentration of free IL-18 does not exceed a few pg/mL (Novick et al., 2001) (Fauteux-Daniel et al., 2023) [3,37]. In a disease state, IL-18 levels rise significantly more than IL-18BP, leading to elevated free IL-18. This makes free IL-18 a major player, and likely the most critical factor, in the associated pathology (Novick et al., 2013) [15].

### 4.2. IL-18opathies

The term IL-18opathies (Landy et al., 2024) [1] was coined for pathologies hallmarked by excessive and unprecedented IL-18 production, most probably a result of a severe imbalance between the levels of IL-18BP and IL-18 (Mazodier et al., 2005) (Girard et al., 2016) (Weiss et al., 2018) (Fauteux-Daniel et al., 2023) [3,34,36,38]. Therefore, solely measuring total levels of IL-18 may sometimes not be sufficiently informative. Free IL-18, the one that is not in a complex with IL-18BP, may thus serve as a key biomarker in the diagnosis of these IL-18opathies and as a criterion in the identification of patients that would benefit from IL-18 blockade. IL-18 levels, 2000–5000 pg/mL, that are at least 10-fold higher than the levels in healthy controls, are suggested to be the cut-off values for the differentiation of systemic juvenile idiopathic arthritis (sJIA) and adult-onset Still’s disease (AOSD) from other pathologies, e.g., Kawasaki Disease, FMF, TRAPS, other subtypes of JIA, SLE, JDM, and leukemia (Shimizu et al., 2022) [39]. The levels of IL-18 in MAS, HLH, and in children with inborn mutations, e.g., mutations in the inflammasome (NLRC4 gain of function) or XIAP (X-linked inhibition of apoptosis) deficiency mutation, are extremely high and are associated with diseases activity. Plasma levels in these pathologies may reach a microgram range of IL-18, namely a range that is 100–1000 higher than in healthy controls (Canna et al., 2014) (Geerlinks & Dvorak, 2022) [40,41]. These findings may justify the inclusion of IL-18 in the international ACR/EULAR classification criteria for these pathologies.

### 4.3. IL-18 in NLRC4-Associated Inflammasomopathies

Inflammasomes are large cytosolic multiprotein complexes of innate immunity. Their components assemble via cytosolic pattern recognition receptors or stress-associated stimuli in response to the detection of infection. This assembly leads to the activation of caspase-1-mediated inflammatory responses, including the cleavage of pro-IL-1β and pro-IL-18 into their active forms, and the initiation of an inflammatory form of cell death, pyroptosis. Inflammasomes consist of a sensor protein, the adaptor protein, an apoptosis-associated speck-like protein containing a caspase-recruitment domain (ASC), and the proinflammatory caspase, caspase-1 (Schroder & Tschopp, 2010) (de Zoete et al., 2014) [42,43]. NLRC4 is one of the components associated with the formation of inflammasome.

Gain-of-function mutations in the gene encoding NLR family CARD domain-containing protein 4 (NLRC4) may result in often-fatal autoinflammatory diseases, the NLRC4-inflammasomopathies, thoroughly reviewed by Romberg et al. (Romberg et al., 2017) [44]. Autoinflammation with infantile enterocolitis (AIFEC) was the first autoinflammation reported that linked IL-18’s major contribution to gut inflammation in myeloid and intestinal epithelial cells. IL-18 level in these patients may reach over 10 microgram levels, 10,000-fold more than in healthy individuals. Additional phenotypes traditionally associated with mutations in another component of the inflammasome NLRP3, like familial cold autoinflammatory syndrome and neonatal onset multisystem inflammatory disease (NOMID), have now also been associated with gain-of-function NLRC4 mutations. These findings highlight the need for specific diagnostic biomarkers and open new therapeutic avenues for treating AIFEC patients (Romberg et al., 2014) (Canna et al., 2014) (Canna et al., 2017) [40,45,46] and the other pathologies with targeted agents like IL-18BP. Children born with the various mutations were safely and successfully treated with recombinant IL-18BP on a compassionate basis for seven years (see Section 6.7.1) and a Phase III clinical trial (NCT03113760) has been recently completed.

### 4.4. IL-18 in XIAP Deficiency

The XIAP gene in humans is located on the X chromosome and the protein encoded by this gene is an inhibitor of apoptosis induced by viral infection and by overproduction of caspases. XIAP is a negative regulator of inflammasome function and represses the production of inflammasome-activated cytokines. Loss of functional mutations in XIAP may lead to life-threatening HLH accompanied by highly elevated IL-18, often 1000-fold higher than a level in healthy controls (Wada et al., 2014) (Geerlinks & Dvorak, 2022) [41,47]. Children born with this mutation were safely and successfully treated with recombinant IL-18BP on a compassionate basis for seven years (see Section 6.7.1) and a Phase III clinical trial (NCT03113760) has been recently completed.

### 4.5. IL-18 in Still’s Disease

The adult and juvenile forms of Still’s disease, AOSD and sJIA, involve extremely high levels of IL-18. AOSD is a rare systemic autoinflammatory disorder of unknown etiology, with an estimated prevalence of 1 in 100,000. This pathology is characterized by a clinical triad of high spiking fever, joint stiffness, and transient skin rash, accompanied by macrophage activation, Th1 cells activation, and an overproduction of IL-1, IL-6, IFN-γ, TNF-a, and particularly IL-18. The latter may reach nanogram and even microgram levels that are 100 to over 1000-fold higher than in other diseases. In a form of MAS characterized by a high mortality rate, 23% of AOSD patients may experience life-threatening complications. It is no surprise that there is a need for diagnostic biomarkers and new treatment options, particularly for cases resistant to conventional therapies. Based on over 100 studies, IL-18 is proposed to be a leading candidate to serve as a diagnostic biomarker in such cases (Canna & De Benedetti, 2024) (Bindoli et al., 2024) (Baggio et al., 2023) (Galozzi et al., 2022) (Shimizu, 2021) (Efthimiou et al., 2021) (Yasin, Fall, et al., 2020) (Kudela et al., 2019) (Giacomelli et al., 2018) [48,49,50,51,52,53,54,55,56].

### 4.6. IL-18 in Differential Diagnosis (HLH and MAS)

Hemophagocytic lymphohistiocytosis (HLH) is a life-threatening, hyper-inflammatory disorder, characterized by multiorgan failure, fever, and cytopenia. Diagnosis of HLH and its subtype MAS remains a challenge. A massive increase in IL-18 blood level is a potential biomarker for HLH/MAS but is currently not a part of diagnostic criteria. The benefit of weaning these patients from steroids and other harsh medications, concomitantly with the stabilization of the disease with IL-18BP, is invaluable. Moreover, if in certain pathologies, e.g., sJIA, high IL-18 levels are detected early enough, it may prevent progression to MAS (Canna & Marsh, 2020) (Krei et al., 2021) [57,58].

### 4.7. IL-18: Predictor of Mortality in Acute Renal Disease

Acute kidney injury (AKI) represents a common and devastating problem in clinical medicine. A major reason is the lack of early biomarkers for AKI, and hence an unacceptable delay in initiating therapy. Urinary interleukin-18 had been reported to be an earlier AKI biomarker than serum creatinine and its level in survivors and non-survivors predicted mortality in patients in an intensive care unit (Chen et al., 2020) (Parikh et al., 2005) (Washburn et al., 2008) [59,60,61]. Mouse models of ischemia-reperfusion have shown that blocking IL-18 prevents renal damage (Wu et al., 2008) [62] and on the other hand, IL-18BP transgenic mice are protected against renal injury (He et al., 2008) [63]. IL-18 also plays a role in kidney transplantation. Of note is a recent study that evaluated changes in the serum concentrations of six IL-1 family cytokines (IL-1 alpha, IL-1 beta, IL-1RA, IL-18, IL-18BP, and IL-36 beta) in 138 kidney allograft recipients vs. healthy donors. In this study, IL-18, and specifically its free form, was the only cytokine that was significantly upregulated )ca. 100-fold, 4000–6000 pg/mL) in the acute rejection group without upregulation of its inhibitor, IL-18BP (Cecrdlova et al., 2024) [64].

Thus, external administration of IL-18BP to selected renal failure patients may prove a kidney-saving and even life-saving therapy.

### 4.8. IL-18 in Acute Respiratory Distress Syndrome

Acute respiratory distress syndrome (ARDS) is yet another devastating pathology in which IL-18 is a player. Mehta et al. has shown that the elevated levels of ferritin in this pathology are mediated by IL-18 and are associated with systemic inflammation and mortality (Mehta et al., 2024) [65]. A multicenter, randomized controlled trial in patients with ARDS, found that, based on canonical classification, one third of patients were misdiagnosed. High levels of IL-18 (≥800 pg/mL) defined a distinct high-risk subgroup associated with mortality. Thus, measurement of IL-18, a marker of inflammasome activity, may provide crucial prognostic information missed by the measurement of traditional inflammatory biomarkers (Moore et al., 2023) [66].

### 4.9. IL-18 in Gastrointestinal System

Based on publications from groups lead by Flavell and by Elinav (Levy et al., 2015) (Nowarski et al., 2015) [67,68], Timothy W. Hand postulated that “IL-18 is the bouncer at the mucosal bar” (Hand, 2015) [69]. An intact intestinal barrier is essential to maintaining a healthy relationship between the host immune system and the microbiota. In 2015, employing conditionally deficient mice in either IL-18 or IL-18 receptor or IL-18BP, Levy et al. and Nowarski et al. displayed the dual nature of IL-18 in the gastrointestinal system. In health, a normal bacterial microbiota produces metabolites, such as taurine, that support the inflammasome-mediated production of IL-18 and anti-microbial proteins in the colon, thus promoting microbial diversity and preventing commensal dysbiosis. In an inflammatory condition, dysbiotic microbiota produce different metabolites, e.g., spermine, that inhibit the inflammasome and inhibit anti-microbial protein production, allowing for its invasive character. During inflammation-induced colitis, IL-18 prevents the development of goblet cells from uncommitted precursors, significantly reducing mucus production and intestinal barrier function. It is therefore suggested that IL-18 targeting may prevent the pathologic breakdown of the mucosal barrier in human ulcerative colitis. IL-18’s function as a safeguard, maintaining a strict equilibrium of epithelial IL-18 signaling, was further demonstrated by Jarret et al. in 2020 (Jarret et al., 2020) [70].

A very recent case report (Guha et al., 2024) [71] presents data on a dramatic improvement and remission of an IBD-associated IL-18opathy in a four-year-old girl that at 6 weeks of age presented with recurrent fevers, rash, and severe gastrointestinal mucosal ulceration. Though the clinical phenotype mirrored the previously reported case of a child born with a mutation in the inflammasome (NLRC4 gain of function gene variant resulting in IL-18 over expression) and who responded to anti-IL-18BP treatment (Canna et al., 2017) [45], in this girl, despite detailed genetic analysis, no known pathogenic variant could be found. The clue to the pathology was found by testing a panel of serum cytokines including IL-1b, TNF-a, IL-6, IFN-α, IFN-γ, IL-10, IL-12, IL-17A, IL-18, IL-23, and IL-33. Only IL-18 was highly elevated. Following two years of hospitalization and a failure of a standard therapy with high dose cortico-steroids and IL-1 inhibitors, remission was achieved by treatment with anti-IL-18 antibody (GSK1070806). Similar to the girl with the NLRC4 mutation, also in this case, the squamous epithelium in the post-esophagus and the colonic mucosa were restored, parenteral nutrition was discontinued and replaced by a normal diet, and the girl was discharged from the hospital. The authors conclude that these cases highlight the importance of measuring IL-18 in patients with autoinflammatory diseases and especially those unresponsive to conventional treatments. It also shows the advantage of blocking IL-18, a treatment associated with no adverse effects, compared to using heavy and potentially harmful medications like steroids.

Another study presents evidence that epithelial inflammasome under certain stress conditions mediates protection against intestinal autoinflammation (Zheng et al., 2023) [72]. Mertens et al. (Mertens et al., 2024) [73] have recently demonstrated this other aspect of IL-18’s dual role, showing that IL-18 may prove beneficial in the gastrointestinal system of mice. Based on their study, these authors postulated that IL-18 programs long-lasting intestinal tolerance via an immune metabolic switch in macrophages. Mediated by IL-18 and confirmed in mice lacking the IL-18 natural antagonist IL-18BP, the switch involves glycolytic polarization through metabolic re-programming to fatty acid oxidation. If applicable, these observations may open personalized therapeutic windows for targeting chronic inflammation in humans.

### 4.10. IL-18 in Skin Diseases

To date, many reports reveal the dysregulation of IL-18 in several skin pathologies, e.g., psoriasis, atopic dermatitis, rosacea, and bullous pemphigoid. Keratinocytes comprise a major part of the epidermis and have a critical role in skin inflammation and immune response. In human keratinocytes, pro-IL-18 is constitutively expressed and is activated to IL-18 upon UV irradiation that in turn affects immune cells, leading to an inflammatory response. Compared to healthy controls, patients with psoriasis exhibit higher levels of IL-18 both in lesions and in blood and the levels correlate with disease severity. As such, IL-18 may serve as a potential biomarker in this pathology. A psoriasis-like mouse model in which an IL-18-neutralizing antibody successfully blocked the harmful Th17 immune response confirmed that indeed, IL-18 is a player in psoriasis. IL-18 was reported to be the most significantly elevated biomarker also in the skin of Atopic Dermatitis (AD) patients and correlated with disease severity (Wang et al., 2023) (Rusiñol & Puig, 2024) [74,75]. Konishi et al. reported that IL-18 transgenic mice developed AD-like dermatitis (Konishi et al., 2002) [76]. Hidradenitis suppurativa (HS) is a chronic skin disease, characterized by clinical inflammation of the hair follicle with the recurrence of abscesses, nodules, and tunnels. Elevated levels of IL-18 and free IL-18 correlating with disease severity were very recently reported by Manzo Margiotta et al. in HS, interestingly with no elevation of IL-1b (Manzo Margiotta et al., 2024) [77].

In view of these findings, blocking IL-18 in skin diseases, either through its natural antagonist IL-18BP or with antibodies, presents a rational therapeutic strategy. Indeed, Jang et al. (Jang et al., 2023) [78] designed a form of IL-18 blockade: APB-R3, a long-acting recombinant human IL-18BP linked to human albumin-binding Fab fragment, SL335. APB-R3 has an extended half-life, reduces liver inflammation and splenomegaly in a mouse model of MAS, and controls skin inflammation in a model of atopic dermatitis.

### 4.11. IL-18 in COVID-19 and in a Cytokine Storm

IL-18 is a player in severe cases of COVID-19. Cytokine storm, a condition described as a highway to hell (Canna & Cron, 2020) (Behrens, 2024) [79,80], is one of COVID’s complications. IL-18 was identified as one of the biomarkers of a cytokine storm signature, including that of COVID-19, and was associated with disease severity (Canna & Cron, 2020) [80]. We (Volfovitch et al., 2022) [81] and others have reported elevated levels of IL-18 in severe cases of COVID-19, with an emphasis on the levels of free IL-18 and IL-18BP (Nasser et al., 2023) (Peleman et al., 2023) (Korotaeva et al., 2024) (Mehta et al., 2024) [65,82,83,84]. Fraser et al. (Fraser et al., 2020) [85] concluded from a study of over 2000 COVID-19 patients that IL-18 is among the top six analytes uniquely elevated in severely ill patients hospitalized in an intensive care unit. A study by Sefik et al. (Sefik et al., 2022) [86] confirmed the role of the inflammasome in general and IL-18 in particular in severe COVID-19. The authors suggest that the replicating SARS-CoV-2 virus in human macrophages activates the inflammasome and initiates an inflammatory cascade, leading to pyroptosis. Pyroptosis is an inflammatory form of lytic programmed cell death, characterized by the activation of caspase-1 and followed by the activation of IL-1, IL-18, and gasdermin. Gasdermin executes pyroptosis via the formation of pores on the cell membrane, leading to membrane rupture and the release of these cytokines (Yu et al., 2021) [87]. Blocking one of the products of the inflammasome with IL-18BP may attenuate the overactive immune-inflammatory response, allowing lung tissue recovery and preventing a transition to fibrosis.

Long COVID affects 60 million people globally, following severe acute respiratory syndrome coronavirus 2 (SARS-CoV-2) infection. Two recent publications (Li et al., 2024) (Krishna et al., 2024) [88,89] point to IFN-γ as a mediator of long COVID and elevated levels of serum IFN-γ are reported in long COVID individuals. Comparative single-cell analysis revealed IFN-γ as a driver of persistent pulmonary inflammation with tissue fibrosis respiratory sequelae after acute COVID-19. Neutralizing IFN-γ after the resolution of acute SARS-CoV-2 infection reduced both these outcomes. The authors propose blocking the IFN-γ signaling axis as a therapeutic intervention, yet they warn that blocking IFN-γ is risky because of the exposure of patients to other infections. Therefore, blocking IL-18, which is an IFN-γ inducing cytokine, but not a master cytokine, may serve as a less risky option and an alternative way of therapy.

### 4.12. IL-18 in Cancer: A Double-Edged Sword

In certain cancer microenvironments, IL-18 is beneficial. IL-18 anti-tumor activity is attributed among others to its ability to increase cytotoxicity and FAS ligand expression (Kaplanski, 2018) [90]. IL-18 has shown anti-tumor activity in different preclinical models of cancer immunotherapy and chemotherapy through the activation of NK and T cell responses. It has been tested in clinical studies in cancer patients and was well tolerated but showed limited efficacy (Robertson et al., 2008) [91]. This limited efficacy is associated with the presence of IL-18’s natural inhibitor, IL-18BP, and its enhancement by IFN-γ in response to IL-18 administration.

Menachem et al. (Menachem et al., 2024) [92] screened over 50 gene-expression studies dealing with myeloid cells in their tumor microenvironment (TME), aiming to identify immune checkpoint proteins suitable for antibody targeting. They have shown that both IL-18 and IL-18BP are upregulated in tumor-associated macrophages (TAMs) but that most of the IL-18 is not in a free form but in a biologically inactive complex with IL-18BP. To restore the activity of endogenous IL-18, they generated COM503, a high-affinity anti-IL-18BP monoclonal antibody, that blocks the interaction between IL-18BP and IL-18 and displaces precomplexed IL-18, thereby enhancing T and NK cell activation. In vivo, this antibody, either alone or in combination with anti-PD-L1 antibody, resulted in tumor growth inhibition, an increase in polyfunctional non-exhausted T and NK cell numbers, and better survival across multiple mouse tumor models. In patients that are resistant to checkpoint immunotherapy, targeting IL-18BP could release IL-18 from its complex, enabling it to exert its beneficial anti-tumor effects.

### 4.13. IL-18 and Immune Checkpoint Inhibitors

Immune checkpoint inhibitor (ICIs) therapy, effective in about 30% of cancer patients, is not an innocent therapy and might cause detrimental, life-long adverse effects. An additional problem that might occur during this treatment is what is termed “pseudoprogression”. Pseudoprogression is not a true tumor escape but a transient inflammatory response and as such requires special attention. Biomarkers that would enable selecting patients that may benefit from ICI therapy are lacking. One such biomarker candidate is IL-18. The rationale behind it is that ICI treatment enhances IFN-γ production, which in turn controls the production of IL-18’s very potent antagonist, IL-18BP. IL-18BP is responsible for the level of the complexed inactive IL-18 and the level of active free IL-18. In addition, it is accepted that the neutrophil-to-lymphocytes ratio serves as a biomarker of response to ICIs and represents the balance between pro-tumoral inflammatory status and anti-tumoral immune response. Indeed, in search of ICI therapy efficacy, Janho Dit Hreich et al. (Janho Dit Hreich et al., 2024) [93] combined the two parameters, IL-18 level and neutrophil concentrations. They performed the study on 195 patients with metastatic non-small cell lung cancer (NSCLC) treated with ICI anti PD-1 monotherapy. Based on the level of circulating IL-18 and combined with neutrophil count, they demonstrated that patients receiving ICI therapy that go into true tumor progression can be distinguished from patients going into “pseudoprogression”. This distinction is very important because the latter should proceed with the ICI therapy while the former need to stop. The authors measured the levels of the three players of the IL-18 system, free IL-18, IL-18BP, and inactive IL-18 that is in complex with its antagonist, IL-18BP. They have confirmed that circulating levels of IL-18 are increased at baseline in NSCLC patients and also showed that compared with true progressors, pseudoprogressors have slightly increased levels of free IL-18. They propose that this difference requires a large-scale validation study to attain statistical significance. They also observe a gradual decrease in the concentration of free IL-18 in patients undergoing ICI therapy. Yet, the main finding of their study is that the level of the inactive IL-18, that is complexed with IL-18BP, is the relevant one to the distinction of the patients with “pseudoprogression” from the patients with a true progression. Moreover, the authors state that patients with high levels of inactive IL-18 are linked to poor overall survival.

IL-18 has been shown to be detrimental in several types of cancer, including advanced gastric cancer, certain subsets of melanomas, and T-cell Acute Lymphoblastic Leukemia. It has demonstrated tumor-promoting effects, such as pro-invasive and pro-angiogenic activities (Fabbi et al., 2015) [19].

### 4.14. IL-18 Enhances CAR-T Treatment

IL-18 warrants special attention in the context of chimeric antigen receptor T-cell (CAR T) therapy in which the patient’s own T cells are engineered and reprogrammed to destroy the patient’s cancer cells. While CAR T therapy’s “claim to fame” is almost exclusively in treating hematological malignancies, it has shown limited success in solid tumors, which make up over 90% of adult cancers. One contributing factor to this limitation points to increased levels of IL-18BP which, due to its exceptionally high affinity to IL-18 and a slow dissociation rate (K_off_) (Kim et al., 2000) [35], binds IL-18 almost irreversibly, effectively removing it from circulation. Zhou et al. (Zhou et al., 2020) [94], from the group of A.M. Ring in Yale (Yale School of Medicine, New Haven, CT, USA), postulated that IL-18BP is a secreted immune checkpoint and barrier to IL-18 immunotherapy in NSCLC, colorectal cancer, and melanoma. This group engineered a recombinant ‘decoy-resistant’ IL-18 (DR-18) that is blind to IL-18BP; namely, it binds IL-18 and transduces the signal but does not bind IL-18BP. The combination of DR-18 and anti-PD-1 therapy produced a synergistic response that resulted in complete colorectal and melanoma tumor regression in mice. To increase the success rate of CAR T therapy in solid tumors, another strategy was worked out in the Memorial Sloan Kettering Cancer Center (Memorial Sloan Kettering Cancer Center, New York, NY, USA). This strategy is aimed at enhancing relevant cytokines production, e.g., IL-18, and thus enabling the T cells to better infiltrate the solid tumor and to improve their specific function in the impaired immunosuppressive tumor microenvironment (Rafiq et al., 2020) [95]. Carl June from the University of Pennsylvania, a pioneer known worldwide for CAR T cell therapy, reviewed these next generation therapies, particularly for solid tumors with an emphasis on brain cancer and including “armoring” CAR T cells with cytokines such as IL-18 (Posey et al., 2024) (Young et al., 2022) [96,97]. Uslu et al. from June’s laboratory showed how to turn immunologically “cold” tumors into “hot” tumors that then become sensitive to CAR-T cell therapy. This group employed IMSA101, a small molecule and an analogue of cGAMP that is an agonist of STING (stimulator of interferon genes) and that induces a pro-inflammatory cytokine milieu in general and IL-18 secretion in particular and thereby enhances CART anti-tumor efficacy (Uslu et al., 2024) [98]. A Phase I clinical study (NCT04684563) by the group of Carl June in patients with non-Hodgkin lymphoma, Chronic Lymphocytic Leukemia, and Acute Lymphoblastic Leukemia, has shown that the product is safe and efficient. A complete response was obtained in six of eight infused patients (Svoboda, 2022) [99].

Additional Phase I clinical studies (NCT05989204 and NCT06287528) were performed in patients with Relapsed or Refractory Acute Lymphoblastic Leukemia to evaluate IL-18 secretion by anti-CD19 CAR T cells engineered to constitutively secrete this cytokine in patients with CD19-positive cancers.

Fischer-Riepe et al. (Fischer-Riepe et al., 2024) [100] employed a similar principle in neuroblastoma and initiated a clinical study (EU CT 2022–501725–21–00). They have engineered GD2IL18CART cells, consisting of CAR-inducible IL-18 along with CAR T cells directed against a surface antigen originating in neuroectodermal tumor, ganglioside GD2. The interaction of GD2-positive tumor cells and the engineered CAR T cells, GD2IL18CART, resulted in higher IFN-γ and TNF-a release and more effective target cytolysis. Promising results had been obtained in a preclinical study and mice treated with cells engineered with the GD2IL18CART construct did not develop tumors.

Studies, in an attempt to improve CAR-T therapy via increase in IL-18 secretion, keep accumulating. Hu et al. (Hu et al., 2017) [101] generated IL-18-secreting chimeric antigen receptor T (IL-18-CAR T) cells to significantly boost CAR T cell proliferation and antitumor activity in melanoma mice. Chmielewski and Abken (Chmielewski & Abken, 2017) [102] engineered IL-18-secreting CAR T cells and named them IL-18 TRUCKs (T cells redirected for universal cytokine-mediated killing). These cells created a cytotoxic and pro-inflammatory environment in advanced tumors and improved the survival of mice with advanced pancreatic and lung tumors. In an automated manner, Glienke et al. (Glienke et al., 2022) [103] scaled up the TRUCK cells to a clinical scale and worked out a procedure for manufacturing a sufficient number of cells for clinical application. These CAR T engineered cells, which released inducible IL-18, exhibited cryopreservation stability and increased cytotoxicity towards target cells carrying the tumor antigen glycosphingolipid GD2.

### 4.15. IL-18 Toxicity in CAR T-Cell Therapy

CAR T-cell therapy toxicity comprises the other edge of the IL-18 sword. In Fischer-Riepe’s very recent study (Fischer-Riepe et al., 2024) [100] attention had been paid to this other aspect of IL-18. Indeed, a proteomics screening in patients before and after treatment with CD19-specific CAR T cells have shown an association between serum IL-18 and immune-cell associated neurotoxicity syndrome (Diorio et al., 2022) [104]. To address this problem of hyperinflammation, Fischer-Riepe et al. (Fischer-Riepe et al., 2024) [100] engineered a construct with a limited systemic release of IL-18 via a strict dependence of this release on the interaction of GD2IL18CART construct with the GD2 target.

The toxicity of CAR T treatment occurs in a subset of CAR T-treated patients and is a result of the life-threatening cytokine release syndrome (CRS) that resembles secondary HLH/MAS. A relatively high incidence of CAR T-cell-associated HLH presenting with highly elevated levels of IL-18 was observed in the CD22 CAR T treatment of relapsing patients or those nonresponsive to CD19-targeted therapies (Lichtenstein et al., 2021) [105]. This suggests a disruption in the endogenous feedback mechanism that regulates biologically active IL-18 levels and calls for preemptive strategies to prevent CRS. IL-18’s natural antidote, IL-18BP, is a major player in this fine-tuned regulation. Based on its efficacy in treating MAS, HLH, and Still’s disease, Tadekinig alfa™ (NCT03113760) is a promising candidate for managing the severe adverse effects or cytokine release syndrome (CRS) in patients undergoing CAR T therapy (Canna et al., 2017) (Geerlinks & Dvorak, 2022) (Gabay et al., 2018) (Yasin, Solomon, et al., 2020) [41,45,106,107].

### 4.16. IL-18 in Other Pathologies

Elevated levels of IL-18 and correlations to diseases activity are reported in rheumatoid arthritis, lupus erythematosus, Wegener’s disease, inflammatory bowel diseases, and cardiac diseases (Novick et al., 2013) [15] and most recently also in Kaposi sarcoma herpes-associated diseases (Lage et al., 2024) [108]. It is evident that IL-18 plays a key role in these pathologies, and its partner in the dyad, IL-18BP, shows promise as a therapeutic drug.

## 5. IL-18 Binding Protein (IL-18BP)

Tight regulation of cytokine-driven signaling, inflammation, and immunoactivation is required to enable counteracting possible deleterious effects such as the transition to chronic inflammation, autoimmunity, fibrosis, and even loss of organ function. The immune system has evolved in such a way that the balance is kept via natural cytokine antagonists operating in a feedback regulation mode of action. Most of these regulators are soluble receptors, while a few are unique binding proteins.

IL-18BP (Novick et al., 1999) [4], osteoprotegerin (OPG), (Simonet et al., 1997) [109], cytokine-like factor-1 (CLF 1) (Elson et al., 1998) [110], and IL-22 binding protein (IL-22BP) (Mühl & Bachmann, 2019) [111] belong to a group of receptor-like decoy proteins and bind the same ligand as the canonical soluble receptor. While canonical soluble receptors are products of either a proteolytic cleavage of the extracellular portion of membrane-bound receptors or of alternative splicing of the corresponding mRNA, the members of the binding family of proteins exist solely in a soluble form and are encoded by a separate gene.

OPG, a member of the TNF receptor family, a physiological antagonist of the receptor activator of NF-κB Ligand (RANKL), and also known as osteoclastogenesis inhibitory-factor (OCIF), is a player in bone-related diseases, cancer, and neurodegenerative disorders and thus is a potential biomarker and a therapeutic target (Baud’huin et al., 2013) (Freeman et al., 2024) [112,113]. Cytokine-like factor 1 (CLF1 or CRLF1) is a secreted receptor belonging to the IL-6 family. CLF1 forms a secreted heterodimer together with its physiologic partner, cardiotrophin-like cytokine (CLC), and this complex is a part of the signaling pathways of ciliary neurotrophic factor receptor (CNTFR), leukemia inhibitory factor (LIFR), and gp130. Mutations in the CLF1 gene are linked to the sometimes-fatal Crisponi or cold-induced sweating syndrome (Kass, 2011) [114]. IL-22BP, a member of the IL-10 cytokine family, is a high-affinity antagonist of IL-22. Though encoded by a separate gene, IL-22BP shares 34% sequence homology with the extracellular domain of IL-22R. IL-22BP guards the dual-natured IL-22 that, depending on the milieu, is either protective or pro-inflammatory. It balances IL-22’s main function in wound healing and in maintaining tissue homeostasis, it may reduce the tumor-promoting activity of IL-22 in hepatocellular carcinoma and may block IL-22 involvement in liver cirrhosis, chronic inflammation, psoriasis, and arthritis (Dumoutier et al., 2001) (Mühl & Bachmann, 2019) (Zenewicz, 2021) [111,115,116].

Though all four members of this unique family of naturally occurring soluble proteins have clinical significance, IL-18BP is the only one that has advanced to clinical trials. Moreover, it has been in compassionate use for seven years now, awaiting FDA approval following a recently completed Phase III clinical study (NCT03113760).

### 5.1. IL-18BP Is Not a Canonical Soluble Receptor

IL-18BP is a rare example of a naturally occurring and extremely potent antagonist. Structurally, it includes a single Ig-like domain with little homology to either chain of the IL-18 receptor. It is unique by virtue of being encoded by a separate gene, located on chromosome 11 (11q13.4), that is distinct from that of the gene coding for the IL-18 cell-bound receptor located on chromosome 2 (2q12.1). IL-18BP therefore deviates from the canonical definition of a soluble receptor. The shared ligand, IL-18, of both the receptor and the binding protein, is located on chromosome 11 (11q22.2). IL-18BP serves as a natural soluble decoy protein and its remarkable affinity for IL-18 prevents the shared ligand from interacting with its cellular receptors, thus making IL-18BP an effective antagonist of IL-18 signaling (Novick et al., 1999) [4].

IL-18BPa binds mature but not pro-IL-18 and is the most active of the four splice variants in humans: IL-18BPa, b, c, and d. IL-18BPc shares the immunoglobulin domain of IL-18BPa, except for the 29 C-terminal amino acids, and the *K*d of IL-18BPc is 10-fold lower than that of IL-18BPa. IL-18BPb and IL-18BPd isoforms lack the entire Ig domain and lack the ability to bind or neutralize IL-18 (Kim et al., 2000) [35]. IL-18BPa is constitutively expressed in hematopoietic and non-hematopoietic cells (Mühl & Bachmann, 2019) [111].

### 5.2. IL-18BP Isolation and Cloning

**Figure 1:**
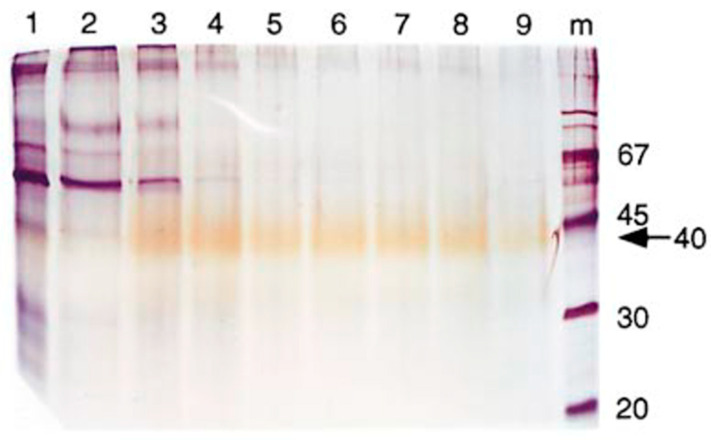
Isolation of IL-18BP: IL-18BP purified from concentrated human urinary proteins by ligand-affinity chromatography on a column of IL-18 covalently bound to Sepharose beads and analyzed by SDS-PAGE and silver staining (Novick et al., 1999) [4]. Lane 1: crude urinary proteins. Lanes 2–9: eight acidic elution fractions from the IL-18 column. Lane m: molecular mass markers (kDa). The arrow indicates the IL-18 binding protein.

Based on our laboratory’s success in isolating the canonical soluble TNF receptors, IL-6 receptor, and both types of interferon receptors (Novick, 2022) (Novick, 2023) [117,118], an attempt was made to isolate the soluble receptor of IL-18. Our strategy of isolation combined two key elements: a rich source of proteins, in our case human urine collected from healthy individuals and concentrated 500-fold, and a simple, specific, and efficient isolation method, ligand (IL-18)-affinity-chromatography (Novick et al., 1999) [4]. A 500 mL batch of concentrated urine (equivalent to 250 L of normal human urine) was applied onto IL-18 (2.5 mg), covalently coupled to a Sepharose-beads (0.5 mL) column, followed by extensive washing and acidic elution of the IL-18-bound proteins (Figure 1). Notably, it was observed that releasing IL-18BP from the affinity column required at least eight column volumes of a highly acidic elution buffer (pH 2.2), whereas all other soluble receptors we previously isolated eluted in a sharp peak within two column volumes, as expected. This “smear” phenomenon hinted at IL-18BP’s exceptionally high affinity for its ligand, which was later confirmed. The pooled elution fractions were subjected to N-terminal sequencing (Edman, 1949) [119] that yielded 40 amino acids. Based on this N-terminal amino acid sequence the corresponding DNA sequence was deduced, and going from protein to DNA, Soo Hyun Kim in our laboratory successfully cloned the IL-18BP. Kim screened cDNA libraries with a probe generated from a 40 N-terminal amino acid sequence of the purified urinary protein. A search in the human cDNA database of the Institute of Genomic Research (TIGR) resulted in an almost perfect match with the cDNA clone THC123801, derived from a human Jurkat T cell library and coding for a protein of unknown function. Interestingly, the transcript was homologous to transcripts encoded by all known Poxviruses, hinting at its function as a potent immunosuppressor. We further characterized the IL-18BP and have shown that the mature IL-18BP protein is a constitutively expressed glycosylated protein composed of 164 amino acids and with a molecular mass of ca. 40 kDa. It blocks the biological activities of IL-18 and inhibits the induction of IFN-γ in vitro and in vivo. Four splice variants were identified, with IL-18BPa being the most abundant and the most active among them (Novick et al., 1999) [4] and IL-18BPa is the subject of this review.

Isolation of the soluble IL-18 receptor failed due to its low affinity to IL-18, and to its low concentration in healthy urine. However, we successfully isolated a second member of a novel family of proteins, the high affinity IL-18BP.

### 5.3. Regulation of IL-18BP

It is well established that IFN-γ, operating through a negative feedback mechanism, is the primary regulator of IL-18BP, and this mechanism is extensively reviewed by Wang (Wang, 2024) [120]. Muhl et al. first demonstrated upregulation of IL-18BP mRNA following an in vitro treatment of keratinocytes, primary renal mesangial cells, and colon carcinoma cells with IFN-γ (Mühl et al., 2000) [121]. Hurgin et al. (Hurgin et al., 2002) [23] then characterized the promoter of IL-18BP and had shown that to avoid premature termination of IL-18 activity, induction of IL-18BP must occur after some delay (details under Section 5.4). Furthermore, using IRF-1-deficient mice, Hurgin et al. confirmed that also in vivo, IFN-γ functions as an IL-18BP inducer, indicating that this pathway is of physiological significance. In addition, IFN-α (Kaser et al., 2002) [122] and IL-27 (Bosmann & Ward, 2013) [123] were demonstrated to stimulate IL-18BP induction. Kaser et al. have shown that IL-18 production is increased in chronic hepatitis C virus-infected patients and that IFN-α therapy of these patients significantly increased IL-18BP plasma levels and substantially reduced the level of free IL-18. IL-27, a member of the IL-12 family, induces IL-18BP in keratinocytes and fibroblasts and its anti-inflammatory function was shown in IL-27 receptor knockout models.

### 5.4. IL-18BP Promoter

Hurgin et al. (Hurgin et al., 2002) [23] in our laboratory characterized the IL-18BP promoter. He identified a gamma-activated sequence (GAS) proximal to the transcription start site, followed by an IFN regulatory factor 1 response element (IRF-E) and two CCAAT enhancer binding protein (C/EBP) sites. Furthermore, he showed that GAS and IRF-E were essential for IFN-γ-induced transcription. IL-18BP is a potent inhibitor of IL-18, an inducer of T helper 1 cytokines such as IFN-γ. Thus, induction of IL-18BP by IFN-γ provides a negative feedback mechanism that shuts off IL-18-elicited immune responses. To avoid premature termination of IL-18 activity, induction of IL-18BP must occur after some delay. Indeed, Hurgin et al. showed that induction was not mediated by the rapid IFN-γ-induced Janus kinase-STAT signaling pathway but rather required de novo synthesis of the transcription factor IRF-1, which together with C/EBP activates the IL-18BP promoter.

### 5.5. IL-18BP Evolutionary Importance

IL-18BP is mimicked by viruses such as Molluscum contagiosum virus and Pox viruses, aiming to evade the host immune system. This phenomenon emphasizes IL-18BP’s critical role in host–pathogen interaction and underlines its evolutionary significance in immune defense mechanisms (Novick et al., 1999) (Smith et al., 2000) [4,124].

### 5.6. IL-18BP Knockout Mice

In 2015, the groups of Flavell and Elinav showed that deletion of the IL-18 gene or its receptor (*Il18r1*) in mouse intestinal epithelial cells conferred protection from colitis and mucosal damage, while the deletion of the IL-18BP gene resulted in severe colitis associated with the loss of mature goblet cells and lethality. They proposed that a strict equilibrium of epithelial IL-18 signaling must be maintained and suggested that IL-18 targeting may prevent the pathologic breakdown of the mucosal barrier in human ulcerative colitis (Nowarski et al., 2015) [68]. Others have shown that IL-18BP is required for normal NK cell maturation, abundance, and function, and that its deficiency results in aberrant proportions of NK cell subsets and increased TNF-a production (Harms et al., 2017) [125]. In yet another mouse model, upon repeated TLR9 stimulation, IL-18BP knockout mice showed severe MAS manifestations, including increased weight loss, splenomegaly, anemia, thrombocytopenia, hyperferritinemia, and bone marrow hemophagocytosis, as well as elevated circulating free IL-18 levels, higher IFN-γ production, and enhanced IFN-γ molecular signature. Therefore, an imbalance between IL-18 and its natural inhibitor IL-18BP may lead to severe MAS, thus reflecting some of the clinical findings of autoinflammatory syndromes such as AOSD and MAS in humans (Girard-Guyonvarc’h et al., 2018) [126].

### 5.7. IL-18BP Affinity and Dissociation Rate: Advantages for Drug Development

The affinity of IL-18BP to IL-18 is very high. Employing a surface plasma resonance, BIAcore, aimed at measuring biomolecular interactions, and using a sensor chip with immobilized human IL-18, we reported a K_D_ of 0.4 nM (Kim et al., 2000) [35]. More recently, a 10-fold higher K_D_ of 0.05 nM was reported (Girard et al., 2016) [34]. This K_D_ is more than two orders of magnitude higher than the affinity of the ligand binding chain of the IL-18 receptor, IL-18Ra, to the same ligand. Moreover, the dissociation rate (K_off_) of the IL-18/IL-8BP dyad is extremely low (Kim et al., 2000) [35], thus making it a very stable complex. Wu et al. (Wu et al., 2003) [33] have shown that the affinity of IL-18BP to IL-18 is comparable to the affinity of IL-18 when bound to both chains of the IL-18 receptor. Notably, in this study IL-18BP demonstrated superior inhibitory activity compared to three neutralizing monoclonal antibodies developed against IL-18. A slow dissociation rate indicates a prolonged binding of a compound to its target, a desirable trait for a prospective drug, as it increases its pharmacokinetics and may even eliminate the need for its modification. Indeed, unlike canonical soluble receptors, e.g., the soluble TNF receptor (Engelmann et al., 1990) [127] that had been translated into the drug Enbrel^®^ as a fusion protein with the Fc portion of immunoglobulin to increase its molecular weight, IL-18BP is an unmodified natural drug. The relatively low molecular weight of IL-18BP is compensated for by its slow dissociation, which allows it to form a very stable complex with IL-18 and prevents rapid clearance by the kidney. Employing a natural, unmodified drug minimizes the risk of provoking an immune response in the patient, thereby decreasing the chances of having to halt the treatment.

## 6. **The Clinical Relevance of IL-18 and IL-18BP**

### 6.1. IL-18BP Level in Health and Disease

Our laboratory generated monoclonal antibodies against IL-18BP and developed a sandwich ELISA aimed to monitor IL-18BP levels in health and disease. In addition, we introduced the term “free IL-18”, IL-18 that is not in a complex with IL-18BP, and most probably it is the level of a free cytokine that dictates the outcome of a pathology. We found that the level of IL-18BP in healthy individuals is around 2 ng/mL and in a disease state the level may rise more than 10-fold (Novick et al., 2001) [37]. In IL-18opathies the increase in the level of IL-18BP is not sufficient for the neutralization of the also increased circulating IL-18 and thus the level of free IL-18 remains high (Novick et al., 2013) [15]. The balance and fine-tuning between IL-18 and IL-18BP is crucial in determining immune responses and disease outcomes. Excessive IL-18 activity, in the absence of IL-18BP, can lead to devastating pathological inflammation and tissue damage, calling for external administration of this natural antidote.

### 6.2. IL-18BP in COVID-19

A study performed on two cohorts of severely ill patients in two hospitals in Washington identified IL-18BP as one of three biomarkers for COVID-19-associated secondary HLH (Canny et al., 2024) [128]. These biomarkers, soluble PDL-1, soluble TNFR1, and IL-18BP, are IFN-inducible proteins and are antagonists of their corresponding ligands. IL-18BP and soluble TNF-R1 have been previously reported to be elevated in subjects with MAS/HLH (Shimizu et al., 2018) (Mazodier et al., 2005) [36,129]. IL-18 in COVID-19 is discussed in Section 4.11.

### 6.3. Fatal IL-18BP Deficiency

Belkaya et al. (Belkaya et al., 2019) [130] reported the death of an 11-year-old girl in France due to fulminant viral hepatitis (FVH) upon infection with hepatitis A virus (HAV). Post-mortem analysis using whole exome sequencing revealed that she had an autosomal recessive single-gene inborn error of immunity, resulting in a complete deficiency of IL-18BP. Both her parents and two siblings were heterozygous for the mutation; therefore, although they were seropositive for HAV, they did not develop FVH. FVH is a life-threatening condition characterized by massive necrosis of the liver, jaundice, encephalopathy, and impaired coagulation, all developing within weeks of the onset of the first symptoms in individuals without preexisting liver disease. IL-18 and IL-18BP are both secreted mostly by hepatocytes and macrophages in the liver. The authors suggest that this patient’s liver had been destroyed by NK and CD8 T cytotoxic cells via the enhanced and uncontrolled IL-18 and IFN-γ-dependent killing of hepatocytes. Based on the success of a treatment with IL-18BP in other conditions in which IL-18 is expressed in excess and based on its beneficial effect in experimental acetaminophen hepatotoxicity (Bachmann et al., 2018) [131] as well as in human acute liver failure due to paracetamol overdosing or liver transplantation, Belkaya et al. proposed that IL-18BP may serve as an antidote in a devastating FVH condition (Belkaya et al., 2019) [130].

### 6.4. The Therapeutic Potential of IL-18/IL-18BP Axis

IL-18 and IL-18BP are associated with various diseases, including autoimmune disorders, inflammatory conditions, infectious diseases, and cancer (Mühl & Bachmann, 2019) [111]. By modulating the IL-18/IL-18BP axis, therapeutic interventions could aim to either enhance or suppress immune responses, depending on the specific disease context. For example, in autoimmune diseases characterized by excessive IL-18 activity, blocking IL-18 or enhancing IL-18BP function could be beneficial. Conversely, in conditions where immune responses are suppressed, such as cancer, strategies to inhibit IL-18BP and boost IL-18 activity might be more appropriate. Indeed IL-18BP had been shown to be a secreted immune checkpoint and barrier to IL-18 immunotherapy (Zhou et al., 2020) [94].

### 6.5. Safety and Tolerability of IL-18BP Therapy

IL-18BP has been translated into the drug Tadekinig alfa and a Phase III clinical trial (NCT03113760) has been recently completed. The studies demonstrated its safety, tolerability, and efficacy profiles. Phase I/II trials, along with seven years of compassionate use of this drug, have consistently demonstrated that IL-18BP therapy is well tolerated and associated with minimal adverse effects. These findings stress the clinical potential of IL-18BP as a safe and effective therapeutic intervention for immune-mediated diseases, making it a promising and low-risk therapeutic option (Tak et al., 2006) (Gabay et al., 2018) (Kiltz et al., 2020) [106,132,133]. The fact that Tadekinig alfa is a self-protein is a significant advantage. Furthermore, its relatively short yet adequate half-life of 2–3 days makes it superior to IL-18-blocking antibodies, which have a half-life of 3–4 weeks. This characteristic is particularly beneficial in situations where treatment must be urgently discontinued to allow IL-18 to perform its role, such as combating a life-threatening infection.

### 6.6. Targeting IL-18

The significance of the IL-18/IL-18BP dyad in various pathologies is reflected in the diverse strategies aimed at targeting IL-18 and IL-18BP, and in the involvement of at least 11 companies in this task. The strategies focus on either blocking or enhancing the relevant players. IL-18’s natural antagonist, IL-18BP, had been translated into a drug, Tadekinig alfa, and anti-IL-18 antibodies have been designed to address cases where IL-18 is detrimental. Conversely, in those cases where IL-18 is beneficial, modified IL-18 with increased biological activity and the ability to evade its decoy protein, is being developed. CAR T cells with dual specificity are engineered in an attempt to potentiate their anti-tumor activity by incorporating an IL-18-producing construct into these cells. Based on the role of IL-18 signaling in tumor immunotherapy, additional innovation combines cancer checkpoint therapy with IL-18 signaling. Variations of PD1-IL18 conjugates are under development, showing a remarkable increase in anti PD-1 potency. Additionally, anti-IL-18BP antibodies are being developed to ensure that IL-18 remains in its active unbound form. A book titled *“Interleukin-18 (IL-18) Inhibitor—Pipeline Insight, 2024”* provides a comprehensive overview of the biology of IL-18, its targeting, and the pipelines of several companies referring to IL-18 therapies. Simcha Therapeutics (New Haven, CT, USA), GlaxoSmithKline (Ontario, Canada), Novartis (Basel, Switzerland), Olatec Therapeutics (New York, NY, USA), Compugen (Tel Aviv, Israel), Lassen Therapeutics (San Diego, CA, USA), Xencor (Pasadena, CA, USA), Bright Peak Therapeutics (Allschwil, Switzerland), Werewolf Therapeutics (Watertown, MA, USA) are among these companies, but the most advanced is AB2 Bio Ltd. (Lausanne, Switzerland) that completed a Phase III clinical study for Tadekinig alfa (IL-18BP). Recombinant IL-18BP that was translated into Tadekinig alfa proved safe and very effective in antagonizing IL-18 and has been in compassionate use for seven years now (Bindoli et al., 2024) [49].

IL-18 is an IFN-γ-inducing factor and member of the IL-1 family. It is a cytokine with a dual nature, participating in both innate and adaptive immunity, but is not a master cytokine. IL-18’s activity is tightly regulated by its highly potent natural antagonist, IL-18BP. IL-18 is mainly a proinflammatory cytokine for host defense against pathogens, but when out of balance with its dyad’s partner, IL-18 is associated with various autoimmune and inflammatory disorders, such as adult-onset Still’s disease, hemophagocytic syndrome, macrophage activation syndrome, fulminant hepatitis, lupus erythematosus, inflammatory bowel diseases, atopic dermatitis, psoriasis, and more. Furthermore, IL-18 is among the cytokines implicated in a cytokine storm that may be a sequalae of autoimmune and inflammatory diseases, viral infections, cancer, and severe cases of COVID-19. IL-18 was also described as an immune checkpoint in cancer treatments such as CAR T cell therapy and anti-PD-1 therapy. Enhancing IL-18 activity in the early stage of immunotherapy and inhibiting it in the late stage proved beneficial. All these features make IL-18 an attractive target for both antagonistic and agonistic approaches. Indeed, academia and industry have taken on the challenge.

### 6.7. IL-18 Inhibitory Drugs 

Drugs under development aimed to inhibit IL-18 activity are listed in Table 1. These include the natural antagonist, IL-18BP, antibodies against IL-18 and an inhibitor to the NLRP3 inflammasome.

#### 6.7.1. IL-18 Binding Protein (Tadekinig Alfa^TM^) by AB2 Bio

Recombinant IL-18BP, the natural antagonist of IL-18, was translated 20 years ago by Ares Serono’s Inc. (now Merck) into the drug Tadekinig alfa. It binds IL-18 with extremely high affinity, forming a very stable complex that effectively neutralizes IL-18’s biological activity. Safety was demonstrated in Phase I and Phase Ib clinical studies, with no observed adverse effects. Judged by the decrease in the level of IFN-γ, Tadekinig alfa was effective in psoriatic arthritis patients chosen for the study, yet the patients did not get better since the duration of the study was too short (Tak et al., 2006) [133]. In 2010, Serono sublicensed Tadekinig alfa to AB2 Bio Ltd. (Lausanne, Switzerland) and several indications such as AOSD, refractory sJIA, and recurrent MAS were considered for a Phase II clinical study. The Phase II clinical study in AOSD patients was successful and awaits Phase III in this indication (Gabay et al., 2018) (Yasin, Fall, et al., 2020) [56,106]. Two devastating pathologies caused by genetic mutations and characterized by an over-expression of IL-18 were chosen for a Phase III clinical study. Phase III was successfully completed in children with a life-threatening inborn mutation in the inflammasome (NLRC4 gain of function) (Canna et al., 2017) [45] and in children with the devastating XIAP deficiency mutation (NCT03113760) (Geerlinks & Dvorak, 2022) (Higuchi et al., 2022) [41,134]. The NLRC4 gain-of-function mutation may result in MAS and a mutation in XIAP may lead to HLH. Treatment with Tadekinig alfa proved lifesaving for these children and years of continuous maintenance remained very effective. Consequently, the FDA approved skipping a Phase II study, allowing direct progression to Phase III trials for these indications. Since 2017, Tadekinig alfa has held FDA orphan drug designation for the treatment of Still’s disease, AOSD, sJIA, and HLH. Moreover, the drug was also approved for compassionate use and over the past seven years, it has kept hospitalizations at bay, enabling these children to lead normal lives. Tadekinig alfa allowed weaning these children from toxic non-specific medications (e.g., steroids) and biologics such as IL-1 and TNF blockers, supporting normal growth and development as well as recovery of their damaged organs.

Tadekinig alfa treatment is being considered for the treatment of MAS, a potential sequalae of cancer and viral diseases, which otherwise has no cure and can have a mortality rate of up to 50%. Another potential candidate for IL-18-blocking therapy is the severe cytokine storm syndrome (Behrens, 2024) [79] seen in conditions such as patients undergoing CAR T therapy (Lichtenstein et al., 2021) (Diorio et al., 2022) [104,105] and in severe cases of COVID-19 (Sefik et al., 2022) [86].

#### 6.7.2. IL-18 Antibody (GSK-1070806) by GlaxoSmithKline

GSK-1070806 is a humanized IgG1/kappa antibody directed against IL-18. It was tested for the treatment of type 2 diabetes, delayed graft function after renal transplantation, and Behcet’s disease and is currently in Phase II clinical study for moderate to severe Atopic Dermatitis (NCT05999799). The study is planned to be completed by 2025.

#### 6.7.3. IL-18 Antibody (Camoteskimab) by Apollo Therapeutics (Avalo Therapeutics and MedImmune)

Camoteskimab (also known as CERC 007, AEVI 007, or MEDI 2338), first developed by Avalo Therapeutics and MedImmune, is a fully human monoclonal antibody which targets IL-18. A Phase I open-label trial was performed in the USA (NCT04752371) and these antibodies are currently being tested in Phase II (NCT06436183) for atopic dermatitis planned to be completed in 2025.

#### 6.7.4. IL-18 and IL-1 Bispecific Antibody (MAS825) by Novartis

*MAS825* is a high-affinity bispecific monoclonal antibody directed against IL-18 and IL-1β. It was tested in COVID-19 pneumonia patients (NCT04382651) but did not meet the primary efficacy endpoint. Nevertheless, in that study, MAS825 antibody combined with standard of care inhibited relevant cytokine pathways, accelerated SARS-CoV-2 virus clearance, and improved impaired respiratory function compared with placebo (Hakim et al., 2023) [135]. Novartis is currently conducting a Phase II clinical study to evaluate the efficacy, safety, and tolerability of MAS825 in indications previously tested by AB2 Bio using Tadekinig alfa (IL-18BP), namely in patients with monogenic IL-18 driven autoinflammatory diseases including NLRC4-GOF, XIAP deficiency, or CDC42 mutations (NCT04641442). The study is anticipated to be completed by 2031.

#### 6.7.5. NLRP3 Inflammasome Inhibitor (Dapansutrile) by Olatec Therapeutics

Inflammasomes, extensively reviewed by Coll and Schroder (Coll & Schroder, 2024) [136], can induce pathological inflammation and tissue damage and are thus a new class of drug targets. One example is Olatec’s oral Dapansutrile (OLT1177^®^) small molecule, β-sulfonyl nitrile. It inhibits the conversion of the inactive pro-IL-1β and pro-IL-18 to their active forms by selectively targeting NLRP3 inflammasome. It is aimed to treat acute and chronic inflammatory diseases such as arthritis, heart failure, asthma, Alzheimer’s disease, Parkinson’s disease, multiple sclerosis, spinal cord injury, type-2 diabetes, melanoma, and breast cancer. OLT1177^TM^ is the only known selective NLRP3 inflammasome inhibitor currently in Phase 2 of clinical trial in the US and Europe.

### 6.8. IL-18 Enhancing Drugs 

Drugs under development aimed to enhance IL-18 activity are listed in Table 2. These include engineered variants of IL-18 resistant to IL-18BP, used elther as monotherapy or combined with anti- PD-1 therapy, IL-18 mimetic agonist impervious to IL-18BP and antibodies targeting IL-18BP.

#### 6.8.1. IL-18 Variant (ST-067) by Simcha Therapeutics

ST-067 is a “decoy-resistant” variant of IL-18, designed to be impervious to IL-18BP. In preclinical studies, ST-067 has shown enhanced anti-tumor immune stimulation. This potential drug is currently in Phase 1/2 clinical study in a variety of solid tumors (melanoma, renal cell carcinoma, triple negative breast cancer, NSCLC, head and neck cancer and more) aimed to test for safety and preliminary efficacy in a form of a monotherapy and in combination with Merck’s checkpoint anti-PD-1 antibody, pembrolizumab (NCT04787042). The study is planned to be completed by 2030 (Zhou et al., 2020) [94].

#### 6.8.2. Modified IL-18 Fused to Fc (XmAb143) by Xencor

IL-18, as a single agent in cancer therapy, exhibits poor pharmacokinetics and an overall lack of efficacy. In attempt to upgrade IL-18’s therapeutic performance, Xencor engineered an IL-18 heterodimeric Fc-fusion protein (XmAb143), with improved thermo-stability, increased half -life from hours to days, and insensitivity to the inhibition by IL-18’s natural antagonist, IL-18BP. Moreover, this modified IL-18 exhibited increased potency in IFN-γ induction by NK and T cells and an over 2000-fold decrease in PD-L1 induction potency as compared to WT IL-18-Fc. XmAb143 exhibited toxicity only in high doses, as demonstrated in pilot studies carried out in cynomolgus monkey (Nisthal, 2022) [137].

#### 6.8.3. Modified IL-18 Fused to Fc and Targeted to PD-1 (BPT 567/PD1-IL18-Fc) by Bright Peak Therapeutics

Bright Peak’s unique protein engineering and chemical conjugation platform is based on the studies of Codarri Deak et al., who developed a new generation of PD-1 cis-targeted cytokine agonists with enhanced therapeutic potential for the treatment of cancer (Codarri Deak et al., 2022) [138]. This strategy enabled the generation of BPT567 conjugate composed of IL-18BP-resistant IL-18 variant with anti-PD-1 antibody, Lipustobart. Following conjugation of the two, BPT567 retained the biologic activity of IL-18 and a functional PD-1/PD-L1 blockade. At the 2024 annual meeting of American Association for Cancer Research (AACR), Bright Peak presented in vitro data showing that BPT567 triggers maximal IFN-γ release in NK92 cells expressing human PD-1, anti PD-1 enhanced potency, and increased IL-18BP resistance due to simultaneous binding to IL-18 receptor and PD-1 on the same cell (cis-signaling). BPT567 exhibits notable single-agent anti-tumor efficacy, superior to that of anti-PD-1 antibody single-agent or non-targeted IL-18. In vivo, BPT567 is well tolerated, induces expansion of effector memory CD8 T cells, and exhibits strong anti-tumor efficacy at significantly lower IL-18 doses compared to the combination of an untargeted antibody-IL-18 conjugate and an anti-PD1 antibody further substantiating the importance of cis-signaling.

#### 6.8.4. Modified and Conditionally Activated IL-18 Resistant to IL-18BP (WTX 518) by Werewolf Therapeutics

Data referring to WTX-518 were presented at the 2024 AACR annual meeting. WTX-518 is being developed to maximize the potential clinical benefit of IL-18 when administered as monotherapy or in combination with checkpoint inhibitors in refractory and/or immunologically unresponsive tumors. WTX-518, an IL-18 pro-drug resistant to IL-18BP inhibition, is conditionally activated within the tumor microenvironment, promotes increased influx and activation of NK cells and polyfunctional CD8 T cells, and induces regressions in mouse tumor models (Morris, 2024) [139].

#### 6.8.5. Antibody Based IL-18 Agonist Resistant to IL-18BP

Lipinski et al. (Lipinski et al., 2023) [140] in collaboration with Merck (Merck Healthcare KGaA, Darmstadt, Germany), employed a strategy that enables the generation of IL-18 mimetics with tailor-made cytokine functionalities aimed at promoting IL-18’s antitumor therapeutic activity. First, they generated bispecific antibody derivatives that mimic the function of IL-18 via cross-linking the IL-18 receptor subunit. This construct was based on single-domain antibodies raised in camelids, specific to IL-18 receptor chains, IL-18 Rα and IL-18 Rβ. They used these antibodies for the screening of yeast surface display and selected variable domains of the heavy chain (VHH), targeting the individual receptor subunits. These bispecific cytokine mimetics constructs were more potent than IL-18 in triggering proinflammatory cytokine release, e.g., IFN-γ, and were unaffected by IL-18 binding protein.

Synthekine further developed this concept, focusing on three key aspects: (1) IL-18 can enhance both innate and adaptive anti-cancer immune responses through signaling across a wide range of cells, (2) IL-18’s proinflammatory activity can counteract the tumor microenvironment’s immunosuppressive state, and (3) IL-18 has shown good tolerance as a single agent in clinical trials. However, IL-18’s efficacy has been limited due to inhibition by IL-18BP. To address this limitation Synthekine engineered IL-18 surrogate cytokine agonists (SCAs). Employing yeast/phage display, Synthekine sorted IL-18 surrogate cytokine agonists composed of the two IL-18 receptor chains’ relevant epitopes that can mimic cytokine signaling. They have shown that the selected SCA expands and activates NK and CD8 positive cells in vitro and in vivo, e.g., IFN-γ induction and NFkB signaling and is not inhibited by IL-18BP.

### 6.9. IL-18 Activity Enhancement Strategy Using IL-18BP Antibodies

#### 6.9.1. IL-18BP Antibody (COM503) by Compugen and Gilead

COM503 is a fully human high-affinity antibody which blocks the interaction between the IL-18 binding protein and IL-18, thereby freeing IL-18 in the tumor microenvironment to inhibit cancer growth. The rationale behind it is that in a certain cancer microenvironment, IL-18 activates anti-tumor effector cells, such as T and NK cells, but the tumor uses IL-18’s natural antagonist, IL-18BP, to block this activity and thus evades the host’s immune system. Compugen employed its proprietary computational discovery platform to identify new drug targets with the ability to enhance anti-cancer immune responses and selected the COM503 anti IL-18BP antibody aimed for the treatment of solid tumors. Compugen is in charge of the ongoing pre-clinical development of COM503 and the Phase 1 study is planned to commence at the end of 2024, while Gilead has the sole right to develop and commercialize COM503. So far, Compugen has shown that COM503 restores human tumor-infiltrating lymphocytes (TILs) and NK cell activity in human assays and that anti-mouse IL-18BP antibody is effective as a monotherapy in mouse breast cancer, colorectal cancer, and melanoma tumor models (Menachem et al., 2024) [92]. Compugen believes that blocking IL-18BP with an antibody represents a promising approach in leveraging cytokine biology in cancer treatment.

#### 6.9.2. IL-18BP Antibody (LASN500) by Lassen Therapeutics

Lassen Therapeutics’ anti IL-18BP antibody is a candidate for human cancer immunotherapy. The company presented preclinical data at the 2023 AACR annual meeting, demonstrating that these antibodies inhibit tumor growth in a mouse syngeneic tumor model. These antibodies also mediate a beneficial proinflammatory response in cancer through enhanced induction of *IFN-γ* and other cytokines, and in combination with the PD-1 antibody they potentiate the activity of IL-18 immunotherapy by inhibiting the checkpoint activity of IL-18BP and enhancing the immune response of NK cells and T cells (Jun et al., 2023 [141]).

### 6.10. IL-18 and CAR T Combined Therapy 

CAR T therapy can be potientiated by cytokines with IL-18 being one of them. Drugs under development aimed to enhance CAR T cell cancer therapy are listed in Table 3. These include engineered CAR T cells constructs that in addition are armored with the ability to secrete IL-18.

#### 6.10.1. IL-18 and CAR T Combined Therapy by Janssen Research & Development

Two basic findings are the rationale and the major catalyst behind IL-18 engineering aimed for CAR T therapy: (1) IL-18 increases the potency of CAR T cells in tumor immunotherapy via the enhancement of T cell proliferation, IFN-γ production, and cytolytic activity, including cytotoxic T-cell and NK-cell-mediated cell killing; (2) in the tumor microenvironment IL-18’s natural inhibitory decoy protein, IL-18BP, reduces IL-18’s activity. The most advanced data on IL-18-armored CAR T cells come from the team led by Carl H. June at the University of Pennsylvania. An ongoing Phase I clinical study is currently being conducted (NCT04684563) by Gilead, employing autologous CAR T cells directed against the human CD19 antigen that also express human IL-18 (HuCART19-IL18). It is known that a significant proportion of patients with relapsed/refractory non-Hodgkin lymphoma (NHL) will not derive a long-term benefit from the existing anti-CD19 CAR T cells. June’s team approached this problem by engineering huCART19-IL18, a 4th generation 4–1BB anti-CD19 construct, armored with the ability to secrete IL-18. So far, it has been shown that treatment with huCART19-IL18 is relatively safe and produces durable remissions in heavily pre-treated lymphoma patients (Svoboda, 2022) (Svoboda, 2024) [99,142].

#### 6.10.2. IL-18 and CAR T Cell Combined Therapy by Eutilex Co., Ltd.

Eutilex Co., Ltd. in Korea engineered a combination of a targeted hepatocellular carcinoma tumor (HCC) marker, Glypican-3 (GPC3), and an IL-18-secreting CAR T cell therapy (EU-307 GPC3-IL18) which is in Phase I and designated for the treatment of HCC and non-small cell lung carcinoma.

#### 6.10.3. IL-18 and CAR T Cell Combined Therapy by the Memorial Sloan Kettering Cancer Center

The Memorial Sloan Kettering Cancer Center is conducting Phase I clinical study (NCT06017258) that tests CAR T cell therapy using a combination of acute myelogenous leukemia (AML) marker and an IL-18-armored construct (CD371-YSNVZIL-18). This study is expected to be completed in 2026.

## 7. Concluding Remarks

The quote from the prophet Amos, “Can two walk together unless meant for each other”, perfectly captures the essence of the IL-18/IL-18BP dyad. This unique biological partnership deserves attention from both basic science and clinical research. The importance of the IL-18/IL-18BP dyad lies in its ability to balance the immune system, making it a potential therapeutic target for autoimmune disorders, inflammatory diseases and cancer, conditions that require precise immune modulation. The rare and distinct features of this dyad make it ideally suited for its regulatory function. IL-18, an IFN-γ inducing factor, and its evolutionary conserved receptor, are IL-1 family members. IL-18 bridges innate and adaptive immunity, is produced by most cells and is always on standby, and induces and is regulated by IFN-γ. It mainly acts as a Th1-associated proinflammatory cytokine but has a dual nature, depending on the microenvironment. Clinical studies have confirmed this duality: IL-18 can be harmful in autoimmune diseases and conditions involving rare mutations, yet beneficial in pathogen defense and certain cancers. IL-18BP is one of only four naturally occurring antagonists that bind the same ligand as cell surface receptors but are encoded by a separate gene. Unlike canonical soluble receptors, these binding proteins, including IL-18BP, lack membrane-bound counterparts. Viral mimicking of IL-18BP suggests its evolutionary significance. Its genetic absence in humans might be fatal. With an exceptionally high affinity for IL-18, much higher than any canonical soluble receptor has to its ligand, IL-18BP controls free IL-18 levels, which are critical in disease. IL-18BP is therapeutically safe and beneficial in various IL-18opathies and has been in compassionate use for many years.

So, what makes the IL-18/IL-18BP dyad special? IL-18, a ready to act cytokine, is a member of the IL-1 family of cytokines, yet is not a master cytokine. It is nevertheless responsible for chronic and acute IL-18opathies, sometimes fatal. To keep IL-18 levels in a normal range, nature has provided a unique, potent, and therapeutically safe antidote, IL-18BP. In the COVID-19 pandemic, this pair, IL-18/IL-18BP, had been reported to play a role in severe cases of the disease. IL-18, recognized as a checkpoint, is an attractive target in one of the most advanced therapies for cancer, CAR-T. These characteristics call for taking advantage of what nature has provided, namely, using IL-18 as an easily measurable biomarker for differential diagnosis, and employing its antidote, IL-18BP, as a safe therapeutic agent. Therefore, IL-18BP should be the primary therapeutic choice for relevant conditions, replacing more aggressive treatments like IL-1 and IL-6 blockers, which carry risks of complications and severe infections. Finally, the dynamic interplay between IL-18 and IL-18BP is context-dependent, with varying effects in different physiological and pathological conditions. While IL-18 promotes inflammation and immune activation, IL-18BP counterbalances it, mitigating excessive IL-18 activity and dampening inflammatory responses. This finely tuned regulation underscores the sophistication of the immune system’s complexity in orchestrating precise immune responses.

## Figures and Tables

**Table 1:** IL-18 inhibitory drugs.

Drug Name	Company	Description	Indications	Clinical Trials	Clinical Trial Number
Tadekinig alfa	AB2 Bio Ltd. (Lausanne, Switzerland)	Recombinant IL-18BP	-NLRC4 mutation -XIAP deficiency	Phase III completed	NCT03113760
Tadekinig alfa	AB2 Bio Ltd. (Lausanne, Switzerland)	Recombinant IL-18BP	AOSD	Phase II completed	NCT02398435
GSK 1070806	GlaxoSmithKline (Ontario, Canada)	Humanized anti-IL-18 IgG1 monoclonal antibody	Atopic Dermatitis	Phase II	NCT05999799
Camoteskimab	Apollo Therapeutics (Cambridge, UK)	Human anti IL-18 IgG1 monoclonal antibody	Atopic Dermatitis	Phase II	NCT06436183
MAS825	Novartis (Basel, Switzerland)	Anti IL-18 and IL-1 Bispecific Antibody	-NLRC4 mutation -XIAP deficiency	Phase II	NLRC4-GOF, NCT04641442
OLT1177 (Dapansutrile)	Olatec Therapeutics (New York, NY, USA)	NLRP3 Inflammasome Inhibitor	Variety of diseases (Section 6.7.5.)	Phase II	EU2020-005227-37

**Table 2:** IL-18 Enhancing Drugs.

Drug Name	Company	Description	Indications	Clinical Trials	Clinical Trial Number or Ref.
ST-067	Simcha Therapeutics (New Haven, CT, USA)	A variant of IL-18 resistant to IL-18BP monotherapy or combined with anti- PD-1	Variety of solid tumors	Phase Ia/II	**NCT04787042**
XmAb143	Xencor (Pasadena, CA, USA)	Modified IL-18 fused to Fc and resistant to IL-18BP	Cancer	Pre-clinical	Nisthal et al., 2022 [137]
BPT 567/PD1-IL18-Fc	Bright Peak Therapeutics (Allschwil, Switzerland)	A conjugate of IL-18 variant resistant to IL-18BP with anti-PD-1 antibody	Cancer	Pre-clinical	Codarri Deak et al., 2022 [138]
WTX 518	Werewolf Therapeutics (Watertown, MA, USA)	Modified and conditionally activated IL-18 resistant to IL-18BP monotherapy or combined with anti-PD-1	Cancer	Pre-clinical	Morris et al., 2024 [139]
SCA	Merck Healthcare KGaA and Synthekine (Darmstadt, Germany and Menlo Park, CA, USA)	IL-18 mimetic agonist resistant to IL-18BP	Cancer	Pre-clinical	Lipinski et al., 2023 [140]
COM503	Compugen and Gilead (Tel Aviv, Israel and Foster City, CA, USA)	Anti-IL-18BP fully human antibody	Mouse breast cancer, colorectal cancer and melanoma	Pre-clinical and Phase I	Menachem et al., 2024 [92]
LASN500	Lassen Therapeutics (San Diego, CA, USA)	Anti-IL-18BP Antibody monotherapy or combined with anti- PD-1	Cancer	Pre-clinical	Jun et al., 2023 [141]

**Table 3:** IL-18 and CAR T Combined Therapy.

Drug Name	Company	Description	Indications	Clinical Trials	Clinical Trial Number or Ref.
HuCART19-IL18	Gilead (Foster City, CA, USA)	Anti CD19 construct secreting IL-18	Lymphoma	Phase I	Svoboda et al., 2022 [99]; Svoboda et al., 2024 [142]
GPC3-IL18	Eutilex Co., Ltd. (Seoul, Republic of Korea)	Targeted hepatocellular carcinoma tumor (HCC) marker, Glypican-3 (GPC3) and an IL-18-secreting CAR T cell therapy	Hepatocellular carcinoma tumor and non-small cell lung carcinoma	Phase I	EU-307 GPC3-IL18
CD371-YSNVZIL-18	Memorial Sloan Kettering Cancer Center (New York, NY, USA)	Combination of acute myelogenous leukemia targeted marker and IL-18-secreting construct	Acute Myeloid Leukemia	Phase I	NCT06017258
